# Social Connectedness as a Therapeutic Tool in Parkinson's Disease

**DOI:** 10.1002/mdc3.70914

**Published:** 2026-11-04

**Authors:** Margherita Fabbri, Lucia Ricciardi, Maria Chiara Sensi, Nicola Modugno, Francesca Morgante

**Affiliations:** ^1^ Department of Clinical Pharmacology and Neurosciences, Parkinson Expert Centre, Centre d'Investigation Clinique CIC1436 University Hospital of Toulouse, NeuroToul COEN Centre, NS‐PARK/FCRIN Network Toulouse France; ^2^ Toulouse Neuroimaging Centre (TONIC), UMR1214 INSERM/UT3 Toulouse France; ^3^ Department of Psychology and Neuroscience, Division of Translational Neuroscience, School of Health and Medical Sciences City St George's University of London, Cranmer Terrace London UK; ^4^ Department of Neuroscience and Rehabilitation Azienda Ospedaliera‐Universitaria S. Anna Ferrara Italy; ^5^ Unit of Neurology IRCCS Neuromed Pozzilli Italy

**Keywords:** Parkinson's disease, social connectedness, social prescribing, well‐being

Living with a chronic and progressing neurodegenerative condition such as Parkinson's disease (PD) can contribute to high levels of loneliness and social isolation. Indeed, more than half of people with PD (PwP) report high levels of loneliness, with depression, social isolation, functional impact of motor symptoms, and male sex identified as key predictors of loneliness in this population.^1^

This viewpoint examines the role of social connection in the lives of PwP, discusses the rationale for using social prescribing (SP) as a potential therapeutic intervention in PD, and presents a real‐world example of its application. Finally, we propose a framework for integrating SP into the care of PwP.

## Social Connection: Definition and Components

Social connection is a multidimensional construct that encompasses the quantity, quality, and variety of interpersonal relationships, together with the individual's perception of belonging, being valued, and receiving support.^2^

Several distinct components contribute to social connection.^2^ Social networks represent the structural dimension, referring to the size, composition, and frequency of interpersonal contacts. Social engagement describes active participation in social, recreational, cultural, or community activities. Social support reflects the functional dimension of relationships and includes emotional, informational, and instrumental assistance provided by family, friends, caregivers, and peers. Finally, sense of belonging captures the subjective perception of being accepted, valued, and integrated within a community. All these components contrast with social isolation, which denotes the objective lack of social contacts and with loneliness, which refers to the distressing gap between desired and perceived social connection. Because these constructs measure different dimensions of social experience, individuals may possess large social networks while still experiencing loneliness, or conversely have relatively few contacts yet remain socially fulfilled. On the other hand, loneliness may reflect, among other dimensions, deficits in close emotional attachment (emotional loneliness) or in broader social relationships and group belonging (social loneliness). Thus, objective social isolation, perceived loneliness, and the different dimensions of social connectedness should be regarded as related but non‐interchangeable constructs.

Social connection is now recognized as a key social determinant of health.^3^, ^4^ Stronger social relationships are associated with reduced morbidity and mortality, improved mental health, healthier behaviors, better cognitive function, and greater resilience to stress, with effects comparable to those of many established cardiovascular risk factors.^5^, ^6^, ^7^

## Why Do People With Parkinson's Need Social Connection?

Social connection is a fundamental component of healthy aging and may be of particular relevance in neurodegenerative disease such as PD whose symptoms may undermine self‐awareness, increase emotional distress, and lead to social withdrawal.^8^

In a prospective analysis of nearly 500,000 UK Biobank participants followed for 15 years, individuals reporting loneliness had a 25% higher risk of developing PD after adjustment for demographic, behavioral, cardiovascular, psychiatric, and genetic risk factors.^9^ Importantly, this association became evident only beyond the first 5 years of follow‐up, suggesting that loneliness may represent a long‐term psychosocial determinant rather than simply an early prodromal manifestation of the disease.

Once PD develops, motor and non‐motor symptoms, together with disease‐related stigma, can progressively impair social participation and erode social connection. Communication difficulties and impaired facial expressiveness further hinder conversations, contributing to misunderstanding, social withdrawal, and loss of interpersonal relationships.^10^ The consequences extend well beyond emotional well‐being. In a survey of more than 1500 PwP, those who reported to be lonely had worse quality of life (QoL) and greater severity of all 33 symptoms measured by a patient‐reported outcome measure.^11^ In a large community‐based study on 3099 subjects, loneliness was a predictor of worse parkinsonism and greater rates of worsening.^12^

These findings suggest that interventions targeting social connectedness have the potential to improve physical and psychological functioning in PwP. However, translating this concept into clinical practice requires a better understanding of which dimensions of social connection can be modified, the most effective interventions and stakeholders to promote them, and robust outcome measures to evaluate their impact.

## What Outcome Are we Aiming to Improve?

Promoting social connection in PwP should ultimately aim to improve outcomes that matter to them, including QoL, well‐being, and happiness.^13^, ^14^ Although these concepts are often used interchangeably, they capture distinct dimensions of health and lived experience.

QoL reflects the perceived impact of the disease on physical, psychological, functional, and social domains and is commonly assessed using disease‐specific patient‐reported outcome measures such as the Parkinson's Disease Questionnaire (PDQ‐39 or its short version PDQ‐8). These measures may not fully reflect broader aspects of living well, such as purpose, meaningful relationships, social participation, or emotional flourishing.^14^ Consequently, interventions that improve social connectedness may have a meaningful impact on patients’ lives without producing substantial changes in QoL.

Well‐being is a broader, multidimensional construct encompassing emotional, psychological, and social functioning. The World Health Organization recognizes well‐being as more than the absence of disease, emphasizing individuals’ ability to realize their potential, cope with life's challenges, work productively, and contribute to their communities.^15^ Well‐being is a multidimensional construct that is challenging to define and measure. Its philosophical roots lie in Aristotle's concept of eudaimonia, which proposed that the highest human good is achieved not through pleasure alone but through living a virtuous and meaningful life and fulfilling one's potential.^16^ The conceptualization of well‐being has evolved considerably in modern psychological research, with contemporary models proposing that well‐being is grounded in the fulfillment of three fundamental psychological needs: autonomy, competence, and relatedness. Within this framework, social connectedness is a key determinant of psychological flourishing and overall well‐being.^17^ More recent models emphasize well‐being as a dynamic balance between an individual's resources and the challenges encountered throughout life.^18^ Hence, well‐being extends beyond symptoms’ control or positive emotions to include purpose, resilience, meaningful relationships, social participation and emotional engagement. This conceptual evolution provides a strong theoretical foundation for the implementation of SP for people with chronic neurological conditions, such as PwP.

Because SP may influence several distinct dimensions of patients’ lives, its effectiveness is unlikely to be adequately captured by a single outcome measure. We therefore propose a multidimensional assessment framework, rather than a fixed battery, encompassing: (1) disease‐related QoL; (2) psychological and eudaimonic well‐being; (3) social connectedness, including loneliness, social networks, social support and sense of belonging; (4) participation in meaningful activities; and (5) personalized, patient‐defined goals. Whenever available, PD‐validated instruments should be prioritized. A recently validated PD social‐connectedness toolkit provides a useful approach to assessing complementary dimensions of social connection and loneliness^19^ while the UCLA Loneliness Scale (ULS‐8) has undergone specific psychometric evaluation in PwP. Table S1 summarizes the candidate measures and related references that could be adopted according to the intervention target and study population.

## Social Prescribing

Social Prescribing (SP) is “the non‐medical prescription of non‐clinical supports and services within the community, typically developed jointly by an individual and a trusted person in a community or clinical setting, with the aim of improving health and well‐being.”^20^ SP enables health care professionals to refer patients to a range of local non‐medical services, including voluntary and community sector organizations, such as local charities and patients’ associations.^21^ Policymakers have started to acknowledge that SP is a component of personalized care. The National Health Service (NHS) in England promotes SP as particularly beneficial for people living with long‐term health conditions, mild mental health difficulties, loneliness or social isolation, and complex social needs affecting physical and psychological well‐being.^22^

SP is increasingly recognized as a meaningful approach to addressing the social determinants of health and as a key component of integrated care, with the potential to reduce healthcare resource utilization, including hospital readmissions.^23^ However, evidence regarding the effectiveness and cost‐effectiveness of SP in primary care and community settings remains mixed, with most studies focusing on its impact on health‐related QoL.^24^, ^25^

A recent review categorized SP interventions into four main areas^26^: (1) increasing social interactions (2) promoting mental and physical well‐being (3) providing instrumental support through occupational therapy/rehabilitation (4) providing home and community care, including home‐based health services and telehealth.

Ideally, SP should be managed by “link workers” (Fig. 1), a professional figure that is funded by NHS England.^27^ In the United States, a similar professional figure is known as a “community health worker” and can be supported through federal programs such as Medicare and Medicaid.^27^ However, in both countries, government funding does not cover the community resources to which patients are referred, creating barriers to equitable access and raising concerns about the sustainability of funding for these services.^28^

**Figure 1:**
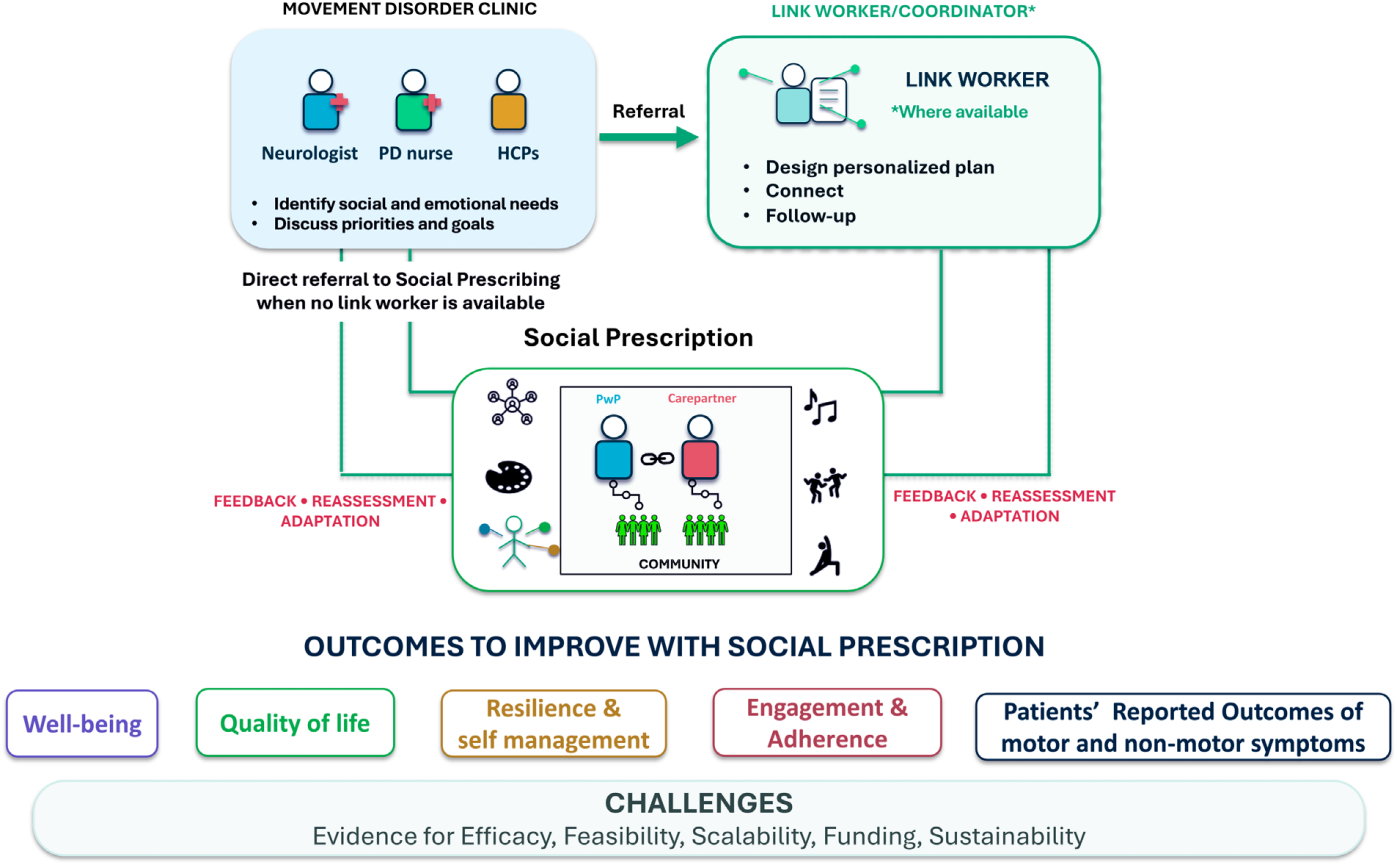
Social connectivity pathway, from the clinic to the community. Social prescribing complements pharmacological and rehabilitative care by linking clinical identification of social needs to personalized community resources. Of note, “link workers” are indicated as an optional step, as their availability across different health‐care system is not known, nor their role well defined.

Additionally, although there is growing evidence of the role of SP in mental health, healthy aging^29^, ^30^ and long term conditions,^31^ a very few studies explored its application in PwP.^32^, ^33^ Subramanian et al introduced the concept of “well‐being prescription” for PwP, defined as “the active pursuit of activities, choices, and lifestyles that lead to a state of holistic health.”^32^

Considering the impact of social isolation in PwP, the potential value of SP may lie in its capacity to target different, and potentially complementary, dimensions of social connectedness. Community activities may expand social networks by creating opportunities for repeated interpersonal contact; structured group activities may promote social engagement through active participation; peer groups and patient organizations may provide emotional, informational and practical social support; and sustained participation in meaningful activities may foster a sense of belonging and shared identity. Importantly, increasing the number of social contacts alone may be insufficient: meaningful connection depends on the quality, continuity and personal relevance of these interactions.^26^ This may partly explain why evidence for SP remains heterogeneous and highlights the importance of matching interventions to individual social needs and preferences. People engagement must therefore be a cornerstone of this process. Rather than passively referring PwP to available activities, SP should involve shared goal‐setting and personalized selection of meaningful activities, followed by reassessment of participation, barriers and individual benefit.^34^, ^35^

People engagement must be the cornerstone of this process, capable of transforming PwP from a passive recipient into a proactive partner of the clinical team, thereby improving adherence, emotional well‐being, and Qol.^36^

However, SP might not be sufficient to improve social connectedness. A recent metanalysis showed that psychological interventions targeting maladaptive social cognitions, alongside interventions facilitating meaningful social interaction and support, may reduce loneliness, although effects vary across intervention types and populations.^37^, ^38^

## Real‐Life Experience: A Social Prescribing Model for PWP

In this section, we highlight real‐world community‐based initiatives showing how social connectedness can become a therapeutic resource. We present the case of ParkinZone (https://www.parkinzone.org), a non‐profit organization operating in Italy since 2006. Building on these real‐world examples, we then propose a framework for integrating social prescribing more systematically into clinical care (Fig. 1).

The Virtual Parkinson Hospital (VPH) is a telemedicine‐based initiative established in 2020 during the COVID‐19 pandemic by a neurologist and four PwP from ParkinZone. The initiative was developed to maintain regular contact and support among PwP, care partners, and healthcare professionals during the pandemic (Video 1). Daily, free‐to‐access sessions in Italian and weekly sessions in English, organized in collaboration with a British patient organization (Spelthorne Parkies), were facilitated by PwP and a group of volunteer neurologists. The program has evolved as an online SP intervention which delivers multiple approaches to promote physical and mental well‐being based on digital interaction, artistic participation, and immersive group experiences.

**Video 1:** Virtual hospital experience during COVID‐19 pandemia.

PwP can self‐refer to VPH or can be referred by their healthcare professionals. The VPH provides approximately 41 sessions per month, engaging around 450 participants monthly. Activities include psychological support groups, theater workshops, patients’ discussion groups held over a “virtual tea,” Tai Chi, Feldenkrais, and the “Question Time” sessions. The latter are held once a week and are attended by approximately 100 PwP and care partners per session. During these sessions, health‐care professionals selected by a steering group composed by PwP, neurologists and psychologists, introduce a topic followed by an hour‐long open discussion. These activities facilitate dialogue, peer support, informal interaction and psychoeducation, creating a social network in which communication and social connectedness becomes part of the therapeutic process.

Importantly, the VPH should not be regarded simply as a digital repository of activities. Its potential value lies in providing repeated opportunities for active and meaningful interaction. Discussion groups may facilitate informal peer relationships; interactive classes and artistic activities promote social engagement; psychological and peer groups provide social support; and recurrent participation in a stable community may contribute to a sense of belonging. Nevertheless, access to such opportunities does not necessarily result in sustained engagement or improved connectedness.^39^ Apathy, depression, cognitive or physical impairment, digital literacy and individual preferences may influence participation. Personalization, active facilitation and repeated reassessment may therefore be as important as the availability of the activities themselves.

Among the activities promoted by ParkinZone, there is also Dance Well. Firstly, developed and supported by the Municipality of Bassano del Grappa (Italy), the project has expanded to become a European Union commissioned project on dance, care, health, and well‐being implemented by six dance organizations and two research institutions in Europe (https://dancewell.eu/en). Dance Well has been founded by the prerequisite of dancing in artistic places, such as museums, theaters, historical buildings, and galleries. Led by professional instructors, Dance Well integrates movement, creativity, and social participation. A pilot trial has provided evidence about its feasibility and safety in PwP with significant improvement of motor, cognitive and emotional outcome measures, including apathy.^40^ By encouraging shared bodily experience and artistic expression, Dance Well helps counteract apathy, emotional withdrawal, and social isolation while fostering motivation, self‐expression, and group cohesion. Since 2020, 63 Dance Well sessions have been organized by ParkinZone with 1062 participants involved (approximately 17/session).

Based on these real‐world experiences in PwP, we propose a personalized, needs‐led social prescribing model embedded within routine clinical care. Clinical teams should identify social and emotional needs and refer patients to a link worker/coordinator, who co‐designs an individualized plan connecting people with appropriate community resources. Ongoing feedback, reassessment and adaptation create a dynamic pathway aimed at sustaining meaningful participation, social connectedness and well‐being (Fig. 1).

## Conclusions

Integrating SP into movement disorder clinic offers an opportunity to complement pharmacological and rehabilitative interventions with person‐centered approaches that address patients’ social and emotional needs and engagement. Successful implementation will require funding of the link‐worker and community resources from government bodies and/or charities, multidisciplinary collaboration, accessible community resources, sustainable healthcare models, and patients and care partners’ engagement facilitated by patients’ associations (Fig. 1). Equally important is the adoption of outcome measures that capture well‐being, social connectedness, participation, and patient‐defined goals. Rather than considering SP as a single intervention, future studies should identify which dimensions of social connectedness are targeted, through which mechanisms, and for whom. Matching individual social needs with meaningful activities and measuring changes across these domains will be essential to determine whether social connectedness can become a genuine therapeutic target in PD.

## Author Roles

All authors have materially participated in the research and/or article preparation (see roles, below). All authors have approved the final submitted article. This article represents original work by the authors, has not been published elsewhere, and is not under consideration for publication elsewhere.

(1) Research project: A. Conception, B. Organization, C. Execution; (2) Statistical Analysis: A. Design, B. Execution, C. Review and Critique; (3) Manuscript Preparation: A. Writing of the first draft, B. Review and Critique.

M.F.: 1C, 2B, 3A.

L.R.: 1B, 2C, 3A.

M.C.S.: 1B, 2C, 3A.

N.M.: 1B, 2C, 3B.

F.M.: 1C, 2B, 3B.

## Disclosures

**Ethical Compliance Statement:** This article is a Viewpoint and does not report original research involving human participants or present new scientific data. Therefore, Institutional Review Board (IRB) or Ethics Committee approval was not required. The authors confirm that they have read the Journal's position on issues involved in ethical publication and affirm that this work is consistent with those guidelines. Written informed consent was not required for the manuscript itself, as it does not report individual patient data or clinical research. The accompanying video includes identifiable people with Parkinson's disease who provided verbal informed consent to be filmed and for the video to be used for educational and scientific dissemination. The video was previously shared with the International Parkinson and Movement Disorder Society as an example of virtual medicine during the COVID‐19 pandemic but has not previously been published. This research did not receive any specific grant from funding agencies in the public, commercial, or not‐for‐profit sectors.

**Financial Disclosures and Conflicts of Interest:** MF: Honoraria to speak from AbbVie, BIAL, Medtronic, Orkyn, Everpharma, Consultancies and advisory board for Teitur Trophics, Zambon and Lundbeck, Grants from MSA Coalition and French Ministry of Health; FM: has received speaking honoraria from Medtronic, Boston Scientific, Teva, Bial, Abbvie, Merz, Ipsen, Zambon. She has been part of advisory boards of Medtronic, Boston Scientific, Bial, Abbvie. She receives Royalties from Springer. F.M. has received financial support for lectures from the International Parkinson's and Movement Disorders Society. She is an associate editor of *Movement Disorders Journal*. LR: has received speaking honoraria from Medtronic, Bial, Abbvie. She has received financial support for lectures from the International Parkinson's and Movement Disorders Society. She is funded by the UKRI Future Leaders Fellowship. MS and NM report no disclosures. Author disclosures are available in the Supporting Information.

## Supporting information


**Data S1.** Disclosure statement form.

**TABLE S1.** Multidimensional assessment framework and candidate outcome measures for studies of social prescribing in Parkinson's disease.

## Data Availability

Data sharing not applicable to this article as no datasets were generated or analysed during the current study.
