# Supplementary material for: Social Connectedness as a Therapeutic Tool in Parkinson's Disease
Source: Mov Disord Clin Pract. 2026 Nov 4:10.1002/mdc3.70914. Online ahead of print. doi: 10.1002/mdc3.70914 (PMC13733433; doi:10.1002/mdc3.70914)
Supplement: Supplementary file 2 [file MDC3-9999-0-s001.docx]

| **DOMAIN** | **CONSTRUCT** | **CANDIDATE OUTCOME MEASURES** | **CONSIDERATIONS IN PD** |
| --- | --- | --- | --- |
| **Health-related quality of life** | Perceived impact of PD on physical, emotional, social and daily functioning | PDQ-39 / PDQ-8¹,² | PD-specific and validated; should be prioritised for disease-related QoL. Their use is supported by reviews of QoL/PROM measures in PD.¹⁶–¹⁸ |
| **Psychological and eudaimonic well-being** | Positive functioning, psychological well-being, meaning, purpose and flourishing | WHO-5 Well-Being Index¹³,¹⁴; PERMA-Profiler³; Ryff Scales of Psychological Well-being⁵; Warwick-Edinburgh Mental Well-being Scale (WEMWBS)⁴ | Captures broader effects potentially missed by disease-specific QoL measures. Instruments should be selected according to the intervention target; PD-specific validation is limited for several measures. |
| **Social connectedness** | Multidimensional assessment of perceived and objective social connection | PD social-connectedness assessment toolkit: brief UCLA Loneliness Scale ULS3); short/long de Jong Gierveld Loneliness Scale; Cohen Social Network Index; brief Perceived Social Support Scale¹⁹ | Recently evaluated specifically in PwP and provides complementary measures of loneliness, social network/isolation and perceived social support.¹⁹ |
| **Loneliness** | Perceived discrepancy between desired and actual social connection; may include social and emotional dimensions | UCLA Loneliness Scale (Version 3)⁸; ULS-3 and de Jong Gierveld Loneliness Scale¹⁹; UCLA Loneliness Scale (ULS-8) ²⁰ | PD-specific psychometric evidence is available for ULS-3/de Jong Gierveld scales¹⁹ and for ULS-8.²⁰ |
| **Social network / isolation** | Size, frequency and structure of interpersonal relationships | Cohen Social Network Index¹⁹; Lubben Social Network Scale (LSNS-6)⁷ | Cohen Social Network Index was evaluated in the PD-specific toolkit.¹⁹ LSNS-6 remains a candidate structural measure, although the cited validation was performed in older adults rather than specifically in PD.⁷ |
| **Social support** | Perceived emotional, informational and instrumental support | Brief Perceived Social Support Scale¹⁹ | The brief Perceived Social Support Scale was evaluated as part of the PD-specific social-connectedness toolkit.¹⁹ |
| **Sense of belonging / perceived connection** | Feeling accepted, valued and connected to others | Social Connectedness Scale-Revised (SCS-R)⁶ | Relevant to the subjective dimension targeted by SP, although PD-specific validation remains limited. |
| **Participation** | Engagement in meaningful social, recreational and community roles | Activity Card Sort (ACS)⁹; Community Integration Questionnaire (CIQ)¹⁰; ICF-based participation measures¹¹ | Particularly relevant to SP because participation may change without parallel changes in conventional clinical outcomes. PD-specific validation should be considered when selecting instruments. |
| **Patient-defined goals and outcomes** | Achievement of personally meaningful goals and changes that matter to the individual | Goal Attainment Scaling (GAS)¹²; Patient-Specific Functional Scale (PSFS)¹⁵; personalised outcome measures | Particularly suited to personalised SP, in which intervention targets differ across individuals. |

**Table S1. Multidimensional assessment framework and candidate outcome measures for studies of social prescribing in Parkinson’s disease.**

QoL: quality of life; PROM: patient reported outcome measure; SP: social prescribing; PERMA: Positive emotion, Engagement, Relationships, Meaning, and Accomplishment.

**References**

1. Jenkinson C, Fitzpatrick R, Peto V, Greenhall R, Hyman N. The Parkinson's Disease Questionnaire (PDQ-39): development and validation of a Parkinson's disease summary index score. Age Ageing. 1997;26(5):353-357.

2. Jenkinson C, Fitzpatrick R, Peto V, Greenhall R, Hyman N. The PDQ-8: Development and validation of a short-form Parkinson's disease questionnaire. Psychology & Health. 1997;12(6):805-814.

3. Butler J, Kern ML. The PERMA-Profiler: A brief multidimensional measure of flourishing. International Journal of Wellbeing. 2016;6:1-48.

4. Tennant R, Hiller L, Fishwick R, et al. The Warwick-Edinburgh Mental Well-being Scale (WEMWBS): development and UK validation. Health Qual Life Outcomes. 2007;5:63.

5. Ryff CD. Happiness is everything, or is it? Explorations on the meaning of psychological well-being. J Pers Soc Psychol. 1989;57(6):1069-1081.

6. Lee RM, Draper M, Lee S. Social Connectedness Scale-Revised. APA PsycTests. 2001.

7. Chang Q, Sha F, Chan CH, Yip PSF. Validation of an abbreviated version of the Lubben Social Network Scale (LSNS-6) and its associations with suicidality among older adults in China. PLoS One. 2018;13(8):e0201612.

8. Russell DW. UCLA Loneliness Scale (Version 3): reliability, validity, and factor structure. J Pers Assess. 1996;66(1):20-40.

9. Baum CM, Edwards D. Activity Card Sort (ACS): Test Manual. 2nd ed. Bethesda, MD: AOTA Press; 2008.

10. Willer B, Ottenbacher KJ, Coad ML. The Community Integration Questionnaire. A comparative examination. Am J Phys Med Rehabil. 1994;73(2):103-111.

11. Resnik L, Plow MA. Measuring participation as defined by the International Classification of Functioning, Disability and Health: an evaluation of existing measures. Arch Phys Med Rehabil. 2009;90(5):856-866.

12. Turner-Stokes L. Goal attainment scaling (GAS) in rehabilitation: a practical guide. Clin Rehabil. 2009;23(4):362-370.

13. World Health Organization Regional Office for Europe. Wellbeing measures in primary health care/the DepCare Project: report on a WHO meeting, Stockholm, Sweden, 12-13 February 1998. 1998.

14. Domenech A, Kasujee I, Koscielny V, Griffiths CEM. Systematic review of the use of the WHO-5 Well-Being Index across different disease areas. Adv Ther. 2025;42(8):3657-3677.

15. Horn KK, Jennings S, Richardson G, Van Vliet D, Hefford C, Abbott JH. The Patient-Specific Functional Scale: psychometrics, clinimetrics, and application as a clinical outcome measure. J Orthop Sports Phys Ther. 2012;42(1):30-42.

16. Martinez-Martin P, Jeukens-Visser M, Lyons KE, et al. Health-related quality-of-life scales in Parkinson's disease: critique and recommendations. Mov Disord. 2011;26(13):2371-2380.

17. Maiuolo ML, Giorgini R, Vaccaro MG, Facchin A, Quattrone A, Quattrone A. Assessment scales for the evaluation of health-related quality of life in Parkinson's disease, progressive supranuclear palsy, and multiple system atrophy: a systematic review. Front Psychol. 2024;15:1438830.

18. de la Cuadra-Grande A, Rejas J, Casado MA, Monroy M, Ruiz M. Quality of life measures in Parkinson's disease: a systematic literature review of patient-reported outcomes measures (PROMs) and their psychometric properties. J Neurol. 2025;272(9):598.

19. González DA, Tosin MHS, Warner-Rosen T, Goetz CG. Quantifying Social Connectedness in Parkinson's Disease: Reliability and Validity of a Clinical Assessment Toolkit. Mov Disord Clin Pract. 2025;12(3):358-363.

20. Subramanian I, McDaniels B, Zhou K, Mischley LK. Psychometric analysis of the short form UCLA Loneliness Scale (ULS-8) in people with Parkinson's disease. Parkinsonism Relat Disord. 2025;135:107828.
